# The Importance of Abdominal Pain in the Elderly: An Unlikely Diagnosis of 17 cm Colo-Colonic Intussusception

**DOI:** 10.7759/cureus.68624

**Published:** 2024-09-04

**Authors:** Thomas Oswald, Sarah Curtis, Malcolm Mckenzie

**Affiliations:** 1 Cardiology, University Hospitals Sussex NHS Foundation Trust, Brighton, GBR; 2 Emergency Medicine, University Hospitals Sussex NHS Foundation Trust, Brighton, GBR

**Keywords:** rcem standards, acute abdominal emergencies, mucinus adenocarcinoma, elderly population, colo-colic intussusception

## Abstract

We report an interesting case of a 17 cm colo-colonic intussusception involving the transverse colon, caecum, and distal small bowel in a previously healthy 79-year-old man. The patient presented to the emergency department with a two-day history of mild, left to right iliac fossa abdominal pain, with no other concerning symptoms. He had a CT of the abdomen and pelvis with contrast for suspected subacute bowel obstruction secondary to recurrent bowel cancer. This was reported as colo-colonic intussusception. Following a surgical review, he was transferred from the local district general hospital for an exploratory laparotomy and underwent a right hemicolectomy with primary ileocolonic anastomosis the same evening. The patient was discharged seven days later following an unremarkable post-operative recovery. Final histology confirmed a mucinous adenocarcinoma of the caecum with postoperative cancer staging as T2N0M0. Following discussion at the colorectal multidisciplinary meeting, no adjuvant therapy was advised, and he was placed on the standard colorectal cancer surveillance program for the next five years.

## Introduction

One of the most common presentations to the ED is abdominal pain, currently estimated to account for between 5-10% of all episodes [1-3]. In the United Kingdom, the Royal College of Emergency Medicine (RCEM) introduced a new standard in 2016, stating that all patients over the age of 70 years presenting with abdominal pain should be reviewed by a consultant in emergency medicine prior to discharge [4]. This particular high-risk group was identified in the national consultant sign-off audit as one of four where consultant review in the ED was recommended. The results demonstrated an average rate of consultant review at 14% in 2017, rising to a rate of 35% across the country when last assessed in 2022 [4]. This shows clear progress in high-risk patient categories receiving review, while demonstrating that improvement is still needed for patient safety. This audit mirrors other international initiatives to improve geriatric patient outcomes, such as geriatric-specific EDs developed by the American College of Emergency Physicians (ACEP) and the creation of the geriatric emergency medicine (GEM) network by the Australasian College for Emergency Medicine (ACEM), aiming to raise awareness and guide training around issues specific to this patient population. Our case explores the complexity of differential diagnosis posed by the presentation of acute abdominal pain in the ED, alongside the difficulty that comes with differentiating appropriate 'need for imaging' decisions taken out of hours. As discussed further, this particular case, which presents the serious, unlikely diagnosis of intussusception in a seemingly well patient with normal initial observations and appearance, highlights the ongoing need for senior review and active discussion among trainees for ongoing education.

## Case presentation

A 79-year-old man presented to the ED with colicky abdominal pain that began 2 days prior, with a pain score of 6/10 at its worst, described as a sharp band across his abdomen from left iliac fossa to right iliac fossa with no radiation. He had no exacerbating factors, and this was the first episode of this type of presentation. He had opened his bowels that day and maintained a regular bowel habit of twice per day until presentation, but described only a 'small amount' in the last 24 hours with no blood visualized. He denied any vomiting, nausea, or fevers, and was passing urine normally. He described his appetite as reduced over the last year and did not believe that he had lost any more weight recently or in the last 6-12 months. He had not traveled out of the country. His primary reason for attending the ED was that his self-administered oral paracetamol was no longer able to control his pain.

His past medical history included bowel cancer 20 years previously, for which he underwent a local resection. He also had essential hypertension, hypothyroidism, and chronic obstructive pulmonary disease. The patient was taking Amlodipine, Levothyroxine, and, when required, salbutamol inhalers. He lived with his wife at home, had stopped smoking 30 years ago, and only consumed alcohol very occasionally, up to three times per year. There was no specific family history of relevance.

Upon arrival in the department, his observations were stable, saturating at 96% on air with a respiratory rate of 20 breaths/minute, a pulse of 75 bpm, blood pressure of 119/64, and a temperature of 36.5 degrees Celsius. He looked comfortable at rest and initially declined pain relief.

On physical assessment, he had a normal cardiovascular and respiratory examination with no peripheral stigmata of chronic disease. On assessment of his abdomen, he had an estimated 15 cm-sized mass lying to the right of the umbilicus. The mass was visible from the end of the bed when the patient lay flat. It was dull to percuss, rigid, and not movable on assessment. On palpation of the mass, he described mild 5/10 pain in severity but was not guarding, and the abdomen was otherwise soft. Bowel sounds were present and of normal intensity on auscultation. There was no organomegaly noted.

Due to the nature of a man in his 70s presenting with acute abdominal pain in the context of a large palpable mass on physical examination, the case was escalated to the on-call emergency medicine registrar who reviewed the patient. After discussion, it was decided to arrange a CT of the abdomen and pelvis with contrast and move the patient to a more observable part of the ED.

Investigations

His initial venous blood gas demonstrated a lactate of 0.8 mmol/L (normal range 0.6-1.8 mmol/L) with no disturbance in his acid-base status, and his initial full blood count showed a hemoglobin of 98 g/L (normal range 135-180 g/L) with no recent baseline recorded for comparison, white blood cells of 10.6 x 10^9^/L (normal range 4-10 x 10^9^/L), neutrophils of 6.8 x 10^9^/L (normal range 2-7 x 10^9^/L), and a biochemistry result for C-reactive protein at 55 mg/L (normal range 0.0-5.0 mg/L).

The focused ultrasound demonstrated an abdominal aorta of 2 cm (normal range <3 cm) and a large, estimated 15 cm, non-fluid-filled mass of undeterminable etiology and differentiation.

**Figure 1:**
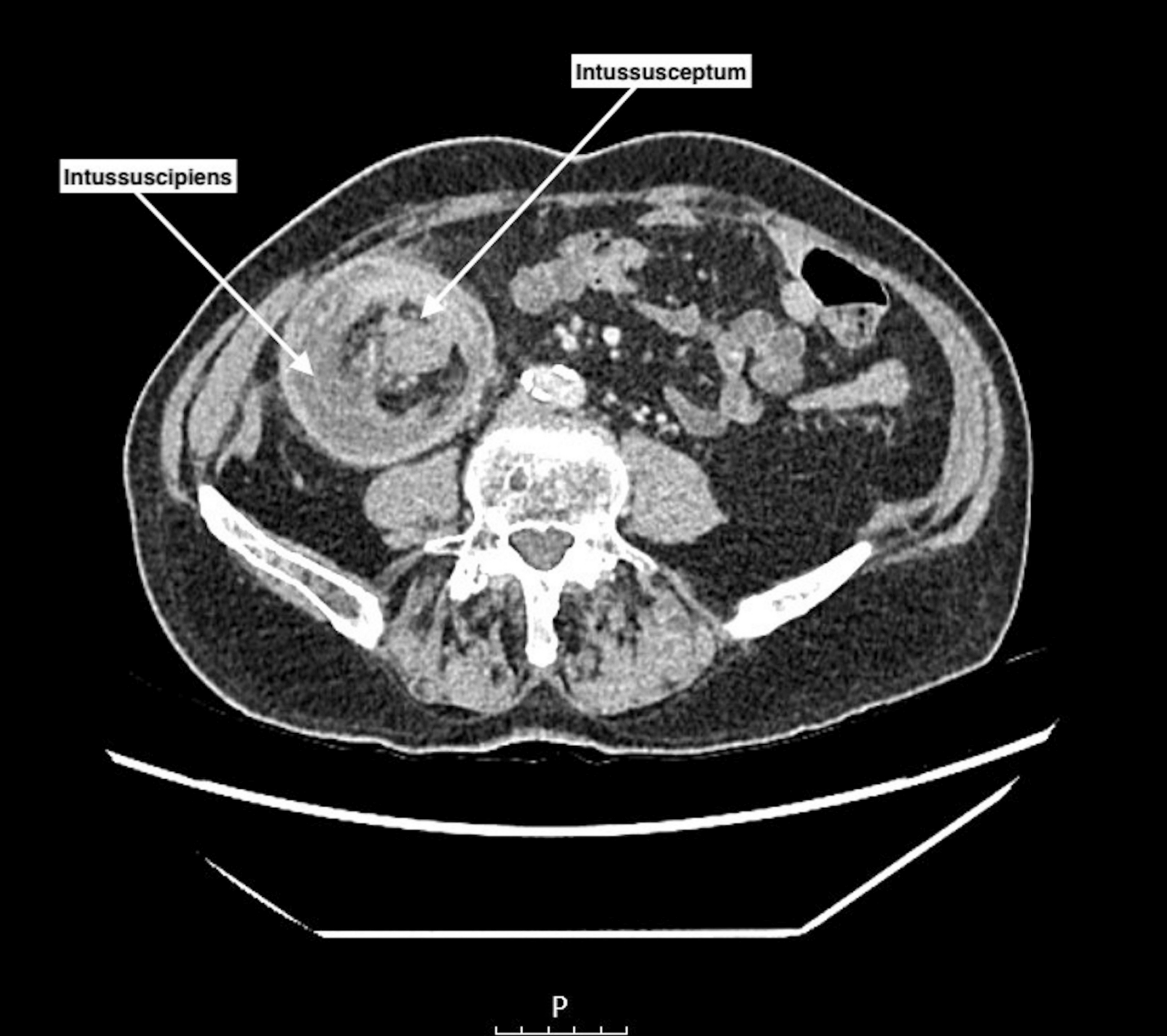
Axial reconstruction of a CT abdomen and pelvis with contrast showing intussusception. Axial CT image shows the 'bowel within bowel' configuration of the proximal part of the intussusception: the intussusceptum centrally is surrounded by mesenteric fat, which is contained within the ring-like intussuscipiens (recipient segment).

**Figure 2:**
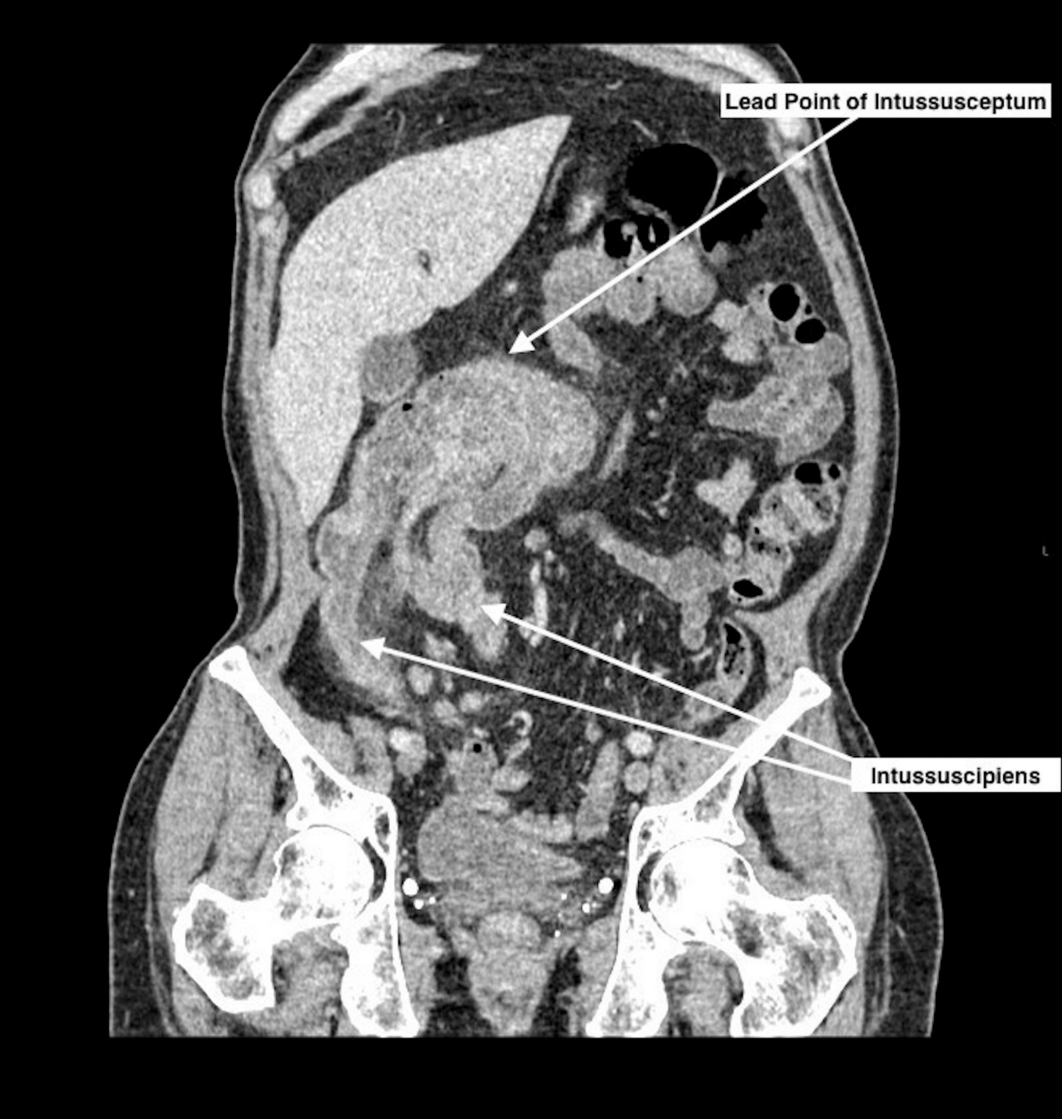
Coronal oblique reconstruction of CT abdomen and pelvis with contrast showing the intussusception. Coronal oblique CT image displaying the bulky lead point of the intussusceptum invaginated into the intussuscipiens.

A CT of the abdomen and pelvis with contrast was performed to assess the origin of the mass (Figures 1-2), demonstrating a 17 cm colo-colonic intussusception involving the transverse colon, the caecum, and the distal small bowel. Additionally, this showed non-dependent linear gas along the bowel wall in keeping with pneumatosis coli, indicative of localized ischemia.

Differential diagnosis 

Intussusception of the bowel is very uncommon in adults and is much more commonly seen in young children with a peak incidence between the ages of 4-36 months. Crucially, this patient had a history of bowel cancer 20 years previously that required surgical resection. The most obvious differentials in this case were adhesions or a bowel cancer recurrence causing a sub-acute bowel obstruction. He mentioned that the amount of feces he was producing was noticeably less than normal within the last 24 hours, representing a subtle change in his bowel habit.

Another factor taken into consideration was that the patient had been unaware of this mass. It was thought to be unlikely that a slow-growing mass that had been increasing in size over a long period would not have been noticed, making volvulus a less likely cause. Given the absence of other past medical history related to inflammatory bowel disease or hematological malignancy, including lymphoma, bowel cancer recurrence as a pathological lead point remained the top differential. This rationale helped determine the need for urgent, "out-of-hours" CT imaging in the context of a remarkably well-seeming patient, as it was felt more likely that what was seen on physical examination was at least manifesting as a more acute process that needed early escalation and surgical review as opposed to an urgent review the following day in same-day emergency care (SDEC).

Treatment 

The patient was initially seen in a district general hospital ED and was then transferred overnight to the local tertiary center with out-of-hours emergency surgical cover, where he underwent a midline exploratory laparotomy with right hemicolectomy of the bowel. He had an ileocolic anastomosis and was started intra-operatively on intra-abdominal sepsis protocol antibiotics (Amoxicillin, Metronidazole, and Gentamicin). Subsequently, the patient was transferred to the High Dependency Unit (HDU) due to a high National Emergency Laparotomy Audit (NELA) risk percentage of mortality at 5.71% but required no further organ support and was therefore stepped down to the surgical ward following a period of observation. His bowel movements were monitored, and his oral diet was slowly re-established before he was deemed medically fit for discharge from the hospital on day seven.

Outcome and follow-up

The case was discussed at the colorectal multi-disciplinary meeting (MDT). The final histology was of a moderately differentiated mucinous adenocarcinoma confined to the muscularis propria with no lymph node spread or venous invasion and with complete surgical margins on excision. The final staging was T2N0M0. The patient did not require any further adjuvant therapy but was entered into the standard colorectal cancer surveillance program involving six-monthly cancer embryonic antigen (CEA) blood tests, six-monthly CT scans, and yearly colonoscopies for a period of five years [1].

The patient was followed up in the surgical clinic three weeks after discharge. He had fully recovered from his surgery and returned to a normal baseline of activity. The outcomes of the MDT, plans for further follow-up, and prognosis were explained to the patient.

## Discussion

The presentation of bowel intussusception is commonly encountered in children, but the approximated incidence in adults is very rare, with estimated case numbers of 2-3 per million presenting in a given year [5]. Bowel intussusception refers to the invagination of a segment of the bowel into the lumen of another segment, which can eventually lead to bowel obstruction and localized ischemia of the GI tract and perforation if not surgically corrected. The most common cause of intussusception in adults is a malignant neoplasm, which represents a pathological lead point. This is in direct contrast to the pediatric population, in which the majority of causes are benign [6]. Of all presentations, the etiology of malignancy accounts for 60% of cases, and given our patient’s history of bowel cancer with previous surgical resection, a recurrence was in keeping with the most probable cause [5].

Due to the scarcity of cases of adult intussusception and the broad spectrum of differential diagnoses that encompasses the presenting complaint of acute abdominal pain, it remains unlikely that emergency clinicians would have this in mind when assessing a patient. Reported durations of symptom onset across a case series by Wang N et al. described 24.4% of presentations as having acute symptoms, 24.4% with subacute symptoms, and 51.2% having chronic symptoms [7]. This places our case in the minority group and further alludes to difficulties in assessment and diagnostic accuracy.

The approach to the geriatric patient with abdominal pain should be more cautious than the equivalent presentation in a young person. It is well understood that this high-risk group presents with atypical symptoms and tends to have multiple comorbidities that can divert attention from the presenting complaint [8,9]. This patient group may be on multiple medications that can mask vital signs, or they may “play down” their experience of symptoms, resulting in unintentional under triage to an area of the ED that deals with more minor ailments [8]. Other psychological factors such as delayed presentation due to a fear of loss of independence and a higher prevalence of cognitive impairment can all affect diagnostic accuracy [8,9].

Our case features a markedly well-looking and behaving patient with normal observations and only marginally raised inflammatory markers on blood tests, presenting with what he described as “minor pain” in the lower abdomen. The decision to perform early CT imaging for geriatric patients presenting with abdominal pain is widely reported as beneficial due to all the confounding factors previously mentioned and has been shown to significantly alter disposition from the ED [10].

We advise a thorough and systematic approach, with early escalation in cases for appropriate consultant review, prompt imaging with CT, and surgical input as needed.

## Conclusions

To conclude, our case explores the scenario of a patient in a high-risk group attending the emergency department with a common presenting complaint while explaining the importance of early escalation to senior clinicians, the benefit of early CT in patient disposition, and highlights an uncommon cause of the acute abdomen in the elderly population. This case demonstrates that the initial clinical picture may not be indicative of the severity of the underlying pathology and that the elderly population with nonspecific symptoms needs comprehensive assessments with an open mind to a broad range of differential diagnoses in cases of uncertainty. The results from the RCEM consultant sign-off audit are promising and suggest that emergency departments are utilizing senior clinical resources in a manner that improves patient safety. Overall, while the diagnosis of intussusception in adults is rare and of significant interest in and of itself, the rapid transition from a well-patient to the need for emergency surgery in a matter of hours is of the most clinical importance for the trainee in emergency medicine.
